# Do Postural and Walking Stabilities Change over a Decade by Aging? A Longitudinal Study

**DOI:** 10.3390/jcm13041081

**Published:** 2024-02-14

**Authors:** Naoki Segi, Hiroaki Nakashima, Sadayuki Ito, Jun Ouchida, Ryotaro Oishi, Ippei Yamauchi, Yuichi Miyairi, Yoshinori Morita, Yasuhiko Takegami, Shinya Ishizuka, Taisuke Seki, Yukiharu Hasegawa, Shiro Imagama

**Affiliations:** 1 Department of Orthopaedic Surgery, Nagoya University Graduate School of Medicine, Nagoya 466-8550, Japan; naoki.s.n@gmail.com (N.S.); sadaito@med.nagoya-u.ac.jp (S.I.); orthochida@gmail.com (J.O.); ryo.oishi@gmail.com (R.O.); yaip0411@yahoo.co.jp (I.Y.); miyairi0317@yahoo.co.jp (Y.M.); bai.an9610@gmail.com (Y.M.); takegami@med.nagoya-u.ac.jp (Y.T.); shinyai@med.nagoya-u.ac.jp (S.I.); imagama@med.nagoya-u.ac.jp (S.I.); 2 Department of Orthopedic Surgery, Aichi Medical University Medical Center, Nagakute 444-2148, Japan; taiseki@aichi-med-u.ac.jp; 3 Department of Rehabilitation, Kansai University of Welfare Sciences, Osaka 582-0026, Japan; hasegawa@tamateyama.ac.jp

**Keywords:** stabilometry, center-of-gravity sway test, standing posture

## Abstract

Background: Previous studies have demonstrated that the center of gravity (COG) is more unstable in the elderly than in young people. However, it is unclear whether aging itself destabilizes the COG. This study aimed to investigate changes in COG sway and gait kinematics over time by a longitudinal study of middle-aged and elderly adults. Methods: This study included 198 healthy middle-aged and elderly people who underwent stabilometry at ten-year intervals. The participants’ mean age at baseline was 62.9 ± 6.5 years, and 77 (39%) of them were male. The results of stabilometry (mean velocity, sway area, postural sway center in the medial–lateral direction [X center], and postural sway center in the anterior–posterior direction [Y center]), and results of exercise tests (the height-adjusted maximum stride length [HMSL] and the 10 m walk test [10MWT]) were analyzed. The destabilized group with 11 participants, whose mean velocity exceeded 3 cm/s after 10 years, was compared with the stable group with 187 participants, whose mean velocity did not exceed 3 cm/s. Results: Mean velocity increased significantly over ten years (open-eye, from 1.53 ± 0.42 cm to 1.86 ± 0.67 cm, *p* < 0.001); however, the sway area did not change significantly. X center showed no significant change, whereas Y center showed a significant negative shift (open-eye, from −1.03 ± 1.28 cm to −1.60 ± 1.56 cm, *p* < 0.001). Although the results of 10MWT and initial HMSL did not differ significantly, the HMSL in the destabilized group at ten years was 0.64, which was significantly smaller than the 0.72 of others (*p* = 0.019). Conclusions: The ten-year changes in COG sway in middle-aged and elderly adults were characterized by a significant increase in mean velocity but no significant difference in sway area. Because the destabilized group had significantly smaller HMSL at ten years, instability at the onset of movement is likely to be affected by COG instability.

## 1. Introduction

Humans should maintain a posture in static standing, responding to postural changes at the onset of movement to a walking state, and maintain dynamic balance during walking.

Even during healthy aging, several age-related changes in the neuromuscular and sensory systems contribute to challenges in maintaining a vertical posture in the field of gravity [1]. A decline in one’s ability to maintain balance is one of the causes of falls [2,3], and the resulting trauma can cause various problems, especially in the elderly, which is why falls in the elderly are considered a social problem, making their prevention a priority [4].

Aging (senescence) considerably affects center-of-gravity (COG) sway in static standing and gait kinematics. Several previous studies conducted using COG sway meters and other devices have already shown that the COG in a standing position is more unstable in the elderly than in young individuals [5,6,7,8,9,10,11]. Additionally, as compared with young adults (30.0 ± 6.1 years), the elderly (74.7 ± 6.6 years) exhibited 17–20% reductions in gait velocity and stride length [12]. Moreover, another previous report demonstrated that the average stride length of the elderly (67.5 ± 3.23 years) was smaller than that of the young (21.4 ± 1.31 years) [13]. However, knowledge of the direct association that exists between COG sway during static standing and gait is limited. Patients with hemiplegia showed a decreasing trend in the area of COG sway with increasing walking ability, although this trend was not statistically significant [14]. However, these cross-sectional studies are insufficient to discuss the association between age-related COG sway and gait kinematics.

This study aimed to investigate the changes in COG sway and gait kinematics over time by a longitudinal study of middle-aged and elderly adults. Additionally, we examined the details of changes in COG sway profiles. Furthermore, we investigated what changes in the exercise tests (e.g., walking ability) were associated with the results of COG sway.

## 2. Materials and Methods

### 2.1. Study Population

This was a retrospective longitudinal study using a prospective database conducted on healthy middle-aged and elderly volunteers who participated in a “basic health checkup” supported by their local government. This medical checkup has been conducted annually in Yakumo-cho, Hokkaido, Japan for more than 30 years, and the local population is already familiar with it. The program for the checkup comprises voluntary orthopedic and physical functional examinations for the inhabitants, and internal medical examinations and psychological tests. An announcement outlining the aims of the health screening program is mailed to the inhabitants every year, and their willingness to participate is confirmed through return mail [15]. As this was an exploratory study, sample size estimation was not possible. Participants who entered the orthopedic and physical functional examinations were included in this study. Thus, the sample consisted mainly of residents who were physically active and independent regarding their daily living activities. Participants who cancelled the stabilometry were excluded. The study was approved by the Human Research Ethics Committee of our institution, and written informed consent was obtained from the participants.

A ten-year longitudinal study was conducted using a dataset recorded for a total of 9939 exercise test participants over a 17-year period (from 2003 to 2019). We included people who underwent stabilometry (the COG sway test) in a given year and again ten years later (for instance, people who underwent stabilometry in both 2003 and 2013). There were 342 such participants, of whom duplicates were excluded and 198 were included in the analysis. The oldest data for the same person were used in the analysis. For example, for people who participated in 2003 and 2013 and 2005 and 2015, only data from 2003 and 2013 were used in the analysis. None of the participants had central or peripheral nervous system dysfunction that could have affected the pattern of stabilometry measurements. Each participant’s age, sex, height, weight, stabilometry results, and exercise test results were analyzed.

### 2.2. Stabilometry (the COG Sway Test)

**Figure 1:**
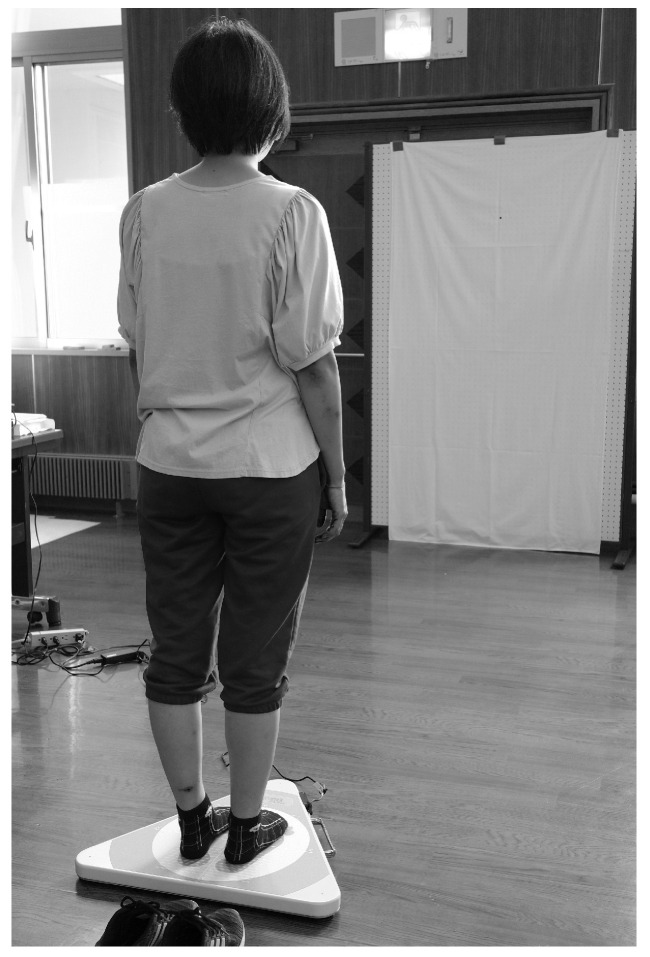
Stabilometry at the basic health checkup.

Postural stability was measured using a G-620 stability force platform (Anima, Tokyo, Japan) designed to evaluate the movement of COP as the COG in the horizontal plane by a triangle force plate equipped with three vertical load sensors [16]. The test environment was set up so that no visual or auditory stimuli were applied to the participants. Participants stood quietly on the footplate without shoes, with arms at their sides and feet close together. The center of both feet was set to be positioned at the center point of the force platform (Figure 1). Participants underwent measurements for 30 s each with open and closed eyes. During the open-eye measurement, participants gazed at a single point in front of them. COP track signals were sampled at a frequency of 20 Hz [15].

Four parameters indicative of postural sway were extracted and stored from the COP time series: (1) mean velocity, (2) sway area, (3) postural sway center in the medial–lateral direction (X center), and (4) postural sway center in the anterior–posterior direction (Y center). Mean velocity represents the average value of the COP sway speed during the measurement time. Sway area represents the inner area enclosed by the outermost outline of the COP track. X center and Y center represent the average position of the COP as seen from the origin. We defined the participants with a mean velocity > 3 cm/s after ten years as the destabilized group, whereas the stable group consisted of all other participants.

### 2.3. Exercise Tests

Exercise function tests that were conducted continuously during the inclusion period were the maximum stride length measurement and the ten-meter walk test (10MWT). Participants were instructed to take as large a step as possible without jumping from a stationary standing position, and this stride length was recorded once for each side. The average of the left and right strides was defined as the maximum stride length, and the maximum stride length (cm) divided by the height (cm) was the height-adjusted maximum stride length (HMSL). Participants were instructed to walk as quickly as possible in a straight line drawn on the floor without running, with appropriate spare sections before and after the ten-meter measurement section. A stopwatch was used to measure the time (s) taken to walk through the measured section, and the results of the 10MWT were recorded.

### 2.4. Statistical Analysis

Data are presented as the mean ± standard deviation. Statistical analyses were performed using R version 4.3.1 (http://www.R-project.org, accessed on 16 June 2023) with Wilcoxon’s rank sum test and Fisher’s exact test, with *p* < 0.05 being considered statistically significant.

## 3. Results

**Figure 2:**
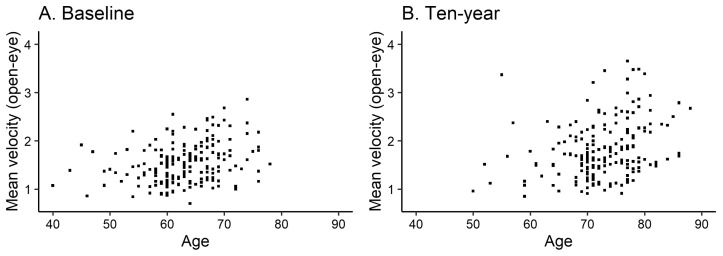
Age and open-eye mean velocity values at baseline (**A**) and ten years after (**B**). Dots are for each sample.

**Figure 3:**
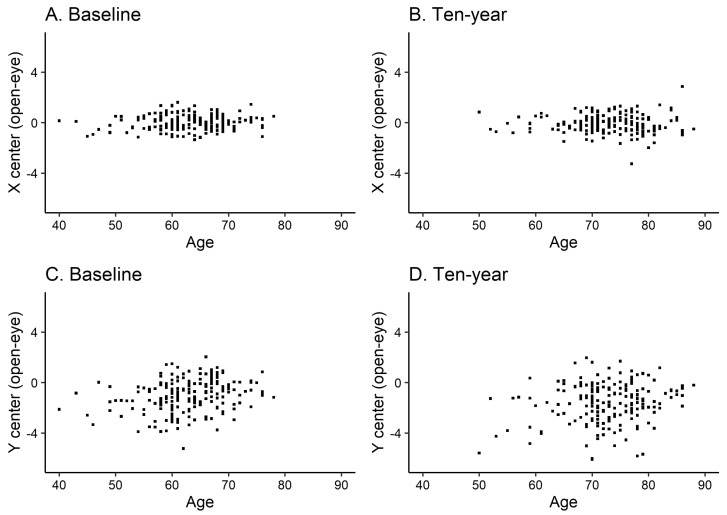
Age and open-eye sway center values at baseline (**A**,**C**) and ten years after (**B**,**D**). X center, postural sway center in the medial–lateral direction; Y center, postural sway center in the anterior–posterior direction. Dots are for each sample.

Table 1 shows the demographic data of all 198 participants at baseline and after ten years for the same individuals. Each participant’s height and weight did not change significantly over the ten-year period. The results of stabilometry were the same for both open-eye and closed-eye assessments, with a significant increase in mean velocity (open-eye, increased from 1.53 ± 0.42 cm/s to 1.86 ± 0.67 cm/s, *p* < 0.001; Figure 2) but no significant change in sway area. Additionally, X center did not change significantly, while Y center was significantly negatively shifted (COP center moved significantly posteriorly) (open-eye, changed from −1.03 ± 1.28 cm to −1.60 ± 1.56 cm, *p* < 0.001; Figure 3). Furthermore, HMSL reduced significantly from 0.74 to 0.72 (*p* = 0.015), while the 10MWT results did not differ significantly.

As the mean velocity results plotted in Figure 2 show, no participant had a baseline mean velocity that exceeded 3 cm/s initially; however, 11 participants had mean velocity values greater than 3 cm/s ten years later. Table 2 shows the results of comparisons between the destabilized group (11 participants, 66.2 years at baseline, 55% men), whose mean velocity exceeded 3 cm/s after 10 years, and the stable group (187 participants, 62.7 years at baseline, 38% men), whose mean velocity did not exceed 3 cm/s. At the time of the initial examination, the destabilized group already had significantly higher mean velocity and sway area values than the stable group. The relationship between the significantly larger mean velocity and sway area remained unchanged after ten years. In contrast, X center and Y center did not differ significantly at baseline or after ten years. Although the results of the 10MWT and the baseline HMSL did not differ significantly, the HMSL in the destabilized group at ten years was 0.64, which was significantly smaller than the 0.72 in the stable group (*p* = 0.019).

## 4. Discussion

The current study of middle-aged and elderly volunteers revealed that humans show significant signs of instability in the COG sway test over time, i.e., aging, in only ten years. In addition, considering inductively that the participants in the destabilized group were significantly older than those in the stable group, it is possible that we continue to be destabilized over time due to the effects of senescence. Furthermore, HMSL was significantly shorter after the ten-year period, and the destabilized group had a significantly shorter HMSL than the stable group. Because HMSL was the span of the first step taken from a stationary standing position, we speculated that the first stability at the onset of movement was affected by the destabilization of the standing position.

The findings of this study are based on the analysis of long-term big data on the general population. This database not only stores a variety of data over a long period of time but also has individuals who have participated multiple times, allowing for longitudinal studies [17]. As the longitudinal data on healthy community-dwelling middle-aged and older adults, including exercise testing, are unique and cannot be replicated elsewhere, this study allowed for a longitudinal analysis of changes in stabilometry results.

A number of previous studies have demonstrated that older adults are more unstable than younger adults in stabilometry [5,6,7,8,9,10,11]. In the proprioceptive system, which plays an important role in controlling balance in the upright posture, the muscle spindle afferents, and the dynamic sensitivity of muscle spindle endings decrease with age [18]. These changes in proprioceptive signals from the lower limb muscles reduce the flexibility of the postural system, ultimately impairing postural control and ability to adapt in the face of changing internal and external conditions [18]. Hasselkus et al. [6] investigated the sway of COG in two postures (the upright stance and forward lean stance) in 10 women aged 21–30 and 73–80 years and found that older adults showed significantly larger sway areas than young adults in both stance positions. The results of such a comparison between young and elderly adults are shown in a meta-analysis by Roman-Liu [10], comparing two groups (adults aged 18–35 and ≥56 years), which showed that the range and velocity of COP were significantly larger in older adults. However, Laughton et al. [5] compared the COG sway of 70 ambulatory community-dwelling older adults and 15 healthy young adults (mean age, 27 ± 3 years) and found that although older fallers had significantly greater anterior–posterior sway during standing than young adults, no significant difference was noted between the older non-fallers and young adults. These results indicate that aging and senescence appear to worsen changes in COG sway, although this does not necessarily occur in everyone.

However, it is noteworthy that these studies compare elderly adults with young individuals in their 20s and 30s who are at the peak of their muscle strength and mobility. The skeletal muscle mass, which directly affects the change in muscle strength with aging, declines a mean of 10% between the ages of 20 and 50 years, and the rate of decline accelerates thereafter, with the elderly losing a mean of 40% of their skeletal muscle mass compared with young individuals [19]. Consequently, healthy elderly people in their 70s–80s have, on average, 20–40% less muscle mass than young people [19]. Thus, because the foundation of the elderly is different from that of young individuals in their 20s and 30s, it is evident that there is a difference in COG sway between the two. Conversely, when considering the health of the elderly, our interest in clinical practice is not so much in the differences between the young and elderly, but rather between the middle-aged and elderly years. Therefore, we conducted a longitudinal study with a dataset of community-dwelling middle-aged and elderly people and demonstrated that COP destabilization does occur in the same individuals over only ten years.

The results of the current longitudinal study showed that aging increases the mean velocity but not the sway area, whereas the Y center becomes more backward. The increase in the mean velocity indicates an increase in COP movement velocity and, therefore, an increase in the total COP movement. Laughton et al. [5], who simultaneously evaluated electromyogram data of young and elderly people found that the elderly had greater muscle activity than young individuals and suggested that high levels of muscle activity are characteristic of age-related declines in postural stability. Our results show consistency with theirs. However, the fact that the sway area has not increased indicates that the range of movement of COP has not changed; in other words, it indicates that the trunk and head remain within a certain range. Surprisingly, Laughton et al. [5] suggest that increased muscle activity in the elderly increases postural sway instead of compensating. Thus, we understand that healthy middle-aged and elderly adults can maintain static standing balance to the extent that body sway increases with age.

Although the fact that the X center remained unchanged and symmetrical was understandable, it is notable that the Y center moved backward. As middle-aged and elderly people tend to tilt their posture forward with increasing age [20,21], our expectation was that their COP would move forward (the Y center would move positively) after 10 years; however, the results were the opposite. We have not been able to determine the reason for this observation; however, it may be indicating the results of postural control strategies of the elderly when maintaining standing postures for such short periods as the stabilometry test lasted for only 30 s.

To understand how the central nervous system (CNS) controls body mechanics, studies of standing posture and balance maintenance have been performed in various scientific disciplines. For example, although simple inverted pendulum models are commonly employed to describe the mechanics of standing, ankle stiffness alone cannot stabilize the standing posture [22]. Detrended fluctuation analysis (DFA) is an analytical method that is supported by physiological evidence and uses a time series analysis approach with intermittent control models. Additionally, an extended DFA analysis of balance and gait has been reported on the basis of the fact that it often shows heterogeneity because of changes in the dynamics of the systems [23]. Although the results are partially consistent with our proposal, the mean velocity of COP essentially reflects the inertial characteristics of the participant rather than the actual changes in balance control by the CNS. Thus, standing balance and gait kinematics are issues that are required to be understood in a more multifaceted manner.

The stability in the standing position may influence the initiation of walking movements. A two-step value of ≤1.3 (approximately 0.65 in terms of HMSL) indicates an increased risk of falling [24]. Thus, the destabilized group had a significantly smaller HMSL and potentially increased risk of falls. The two-step test, which is similar to HMSL, measures the stride length for assessing walking ability, including the muscle strength, balance, and flexibility of the lower limbs [25], and has been incorporated into the diagnosis of locomotive syndrome. Locomotive syndrome, which is a concept that was proposed by the Japanese Orthopaedic Association in 2007, refers to a state of reduced motor function because of age-related musculoskeletal disorders [26]. As the multi-joint coordinated movements of the lower extremity joints are required for standing balance and walking [27], the movements that can be safely produced from a state of lower COP stability should be smaller. Therefore, it is possible that the destabilized group had significantly smaller HMSL values because they could not take a stable first step and because of the potentially increased risk of falling. However, the 10MWT results did not deteriorate significantly over the 10-year period. Although a decrease in walking speed in the elderly is important because it is associated with an increased risk of falls [28], there may be no association between COP instability experienced by healthy middle-aged and elderly individuals over time and their maximal walking speed. Hence, the association between walking and COP instability remains an issue to be investigated in the future.

Several studies have been conducted for the prevention of balance and gait disorders in older adults. A review of randomized controlled trials targeting fall prevention in elderly living in a community [29] found that exercise programs can reduce the rate of falls and number of individuals experiencing falls, and exercise programs that reduce falls primarily included balance and functional exercises. Additionally, the multicomponent exercise interventions consisting of strength training, endurance training, and balance training improve the fall rates, walking ability, balance ability, and strength performance in frail older adults [30]. Although it is difficult to determine directly applicable measures from the current study, the measurement of the maximum stride length in community-dwelling older adults may provide an estimate of balance and walking ability and may trigger interventions to improve their standing balance and gait stability.

Our study had several limitations. First, the number of subjects included was small since this was a longitudinal study with a ten-year study period. Second, the participants in the destabilized group were significantly older than those in the stable group. However, because this difference was only 3.5 years on average, we argued that the effects of age differences on our results were probably limited. Third, as the raw stabilometry data were not stored, the analysis of the COP time series requiring raw data was not possible. Despite these limitations, we were able to characterize how the results of the stabilometry varied over the ten-year period of this longitudinal study.

## 5. Conclusions

We investigated the changes in COG sway in community-dwelling middle-aged and older adults through a ten-year longitudinal study. After 10 years, the COP mean velocity showed a significant increase. The destabilized group had greater mean velocity and sway area values at the initial examination than the stable group, suggesting that there may be a threshold at which COP becomes unstable with age. Furthermore, the destabilized group had significantly smaller HMSL at 10 years than the stabilized group. Thus, instability at the start of movement from the standing position may be influenced by instability of the COP.

## Figures and Tables

**Table 1:** Baseline and ten-year demographic data.

	Baseline [95% CI] N = 198	Ten-Year [95% CI] N = 198	Difference of Means	*p*-Value
Age, year	62.9 ± 6.5	72.9 ± 6.9		<0.001
Sex, men	77 (39%)	77 (39%)		
Height, cm	156.3 ± 7.8	155.0 ± 7.6	−1.1 ± 3.2	0.14
Weight, kg	57.0 ± 8.6	55.9 ± 8.9	−1.0 ± 5.0	0.23
BMI, kg/cm^2^	23.3 ± 2.8	23.2 ± 3.0	−0.1 ± 1.8	0.90
Open-eye stabilometry				
Mean velocity, cm/s	1.53 ± 0.42 [1.5, 1.6]	1.86 ± 0.67 [1.8, 2.0]	0.33 ± 0.51	<0.001
Sway area, cm^2^	2.58 ± 1.54 [2.4, 2.8]	2.81 ± 1.53 [2.6, 3.0]	0.23 ± 1.54	0.075
X center, cm	0.00 ± 0.58 [−0.08, 0.08]	−0.08 ± 0.72 [−0.18, 0.02]	−0.08 ± 0.74	0.24
Y center, cm	−1.03 ± 1.28 [−1.2, −0.85]	−1.60 ± 1.56 [−1.8, −1.4]	−0.57 ± 1.65	<0.001
Close-eye stabilometry				
Mean velocity, cm/s	2.07 ± 0.72 [2.0, 2.2]	2.41 ± 1.04 [2.3, 2.6]	0.34 ± 0.79	0.004
Sway area, cm^2^	3.13 ± 1.83 [2.9, 3.4]	3.53 ± 2.18 [3.2, 3.8]	0.40 ± 2.16	0.12
X center, cm	0.00 ± 0.61 [−0.08, 0.09]	0.03 ± 0.70 [−0.07, 0.13]	0.03 ± 0.72	0.65
Y center, cm	−0.29 ± 1.25 [−0.46, −0.11]	−1.09 ± 1.70 [−1.3, −0.85]	−0.80 ± 1.60	<0.001
Exercise Tests				
10MWT, s	5.15 ± 0.79 [5.0, 5.3]	5.36 ± 1.06 [5.2, 5.5]	0.22 ± 0.94	0.075
HMSL	0.74 ± 0.08 [0.73, 0.75]	0.72 ± 0.08 [0.71, 0.73]	−0.03 ± 0.08	0.015

BMI, body mass index; X center, postural sway center in the medial–lateral direction; Y center, postural sway center in the anterior–posterior direction; 10MWT, 10 m walking test; HMSL, height-adjusted maximum stride length.

**Table 2:** Comparison between the destabilized and stable groups at baseline and ten years after.

	Baseline			Ten-Year		
	Destabilized [95% CI] N = 11	Stable [95% CI] N = 187	*p*-Value	Destabilized [95% CI] N = 11	Stable [95% CI] N = 187	*p*-Value
Age, year	66.2 ± 8.1	62.7 ± 6.3	0.019	76.4 ± 8.2	72.7 ± 6.5	0.018
Sex, men	6 (55%)	71 (38%)	0.34	6 (55%)	71 (38%)	0.34
Height, cm	159.2 ± 10.0	156.1 ± 7.7	0.18	157.91 ± 10.81	154.82 ± 7.43	0.28
Weight, kg	59.6 ± 7.7	56.8 ± 8.7	0.18	60.3 ± 6.6	55.6 ± 9.0	0.081
BMI, kg/cm^2^	23.6 ± 2.8	23.3 ± 2.8	0.69	24.3 ± 3.2	23.2 ± 3.0	0.44
Open-eye stabilometry						
Mean velocity, cm/s	2.21 ± 0.40 [1.9, 2.5]	1.49 ± 0.38 [1.4, 1.5]	<0.001	3.73 ± 0.58 [3.3, 4.1]	1.75 ± 0.50 [1.7, 1.8]	<0.001
Sway area, cm^2^	4.24 ± 2.04 [2.9, 5.6]	2.48 ± 1.45 [2.3, 2.7]	0.002	5.71 ± 1.23 [4.9, 6.5]	2.63 ± 1.37 [2.4, 2.8]	<0.001
X center, cm	0.15 ± 0.66 [−0.30, 0.59]	−0.01 ± 0.57 [−0.09, 0.07]	0.39	0.13 ± 0.70 [−0.34, 0.60]	−0.09 ± 0.72 [−0.20, 0.01]	0.37
Y center, cm	−0.64 ± 1.32 [−1.5, 0.25]	−1.05 ± 1.28 [−1.2, −0.87]	0.15	−0.81 ± 1.44 [−1.8, 0.16]	−1.65 ± 1.56 [−1.9, −1.4]	0.064
Close-eye stabilometry						
Mean velocity, cm/s	3.04 ± 0.80 [2.5, 3.6]	2.02 ± 0.67 [1.9, 2.1]	<0.001	4.96 ± 0.83 [4.4, 5.5]	2.26 ± 0.84 [2.1, 2.4]	<0.001
Sway area, cm^2^	4.50 ± 2.39 [2.9, 6.1]	3.05 ± 1.77 [2.8, 3.3]	0.019	7.27 ± 1.78 [6.1, 8.5]	3.30 ± 1.99 [3.0, 3.6]	<0.001
X center, cm	0.35 ± 0.63 [−0.07, 0.78]	−0.02 ± 0.60 [−0.11, 0.07]	0.083	0.24 ± 0.83 [−0.32, 0.80]	0.02 ± 0.70 [−0.08, 0.12]	0.36
Y center, cm	0.34 ± 1.52 [−0.68, 1.4]	−0.32 ± 1.23 [−0.50, −0.15]	0.10	−0.11 ± 1.91 [−1.4, 1.2]	−1.14 ± 1.68 [−1.4, −0.90]	0.10
Exercise Tests						
10MWT, s	5.25 ± 1.09 [4.5, 6.0]	5.14 ± 0.77 [5.0, 5.3]	0.94	5.23 ± 1.06 [4.5, 6.0]	5.36 ± 1.06 [5.2, 5.5]	0.92
HMSL	0.72 ± 0.11 [0.64, 0.79]	0.74 ± 0.07 [0.73, 0.75]	0.53	0.64 ± 0.09 [0.55, 0.73]	0.72 ± 0.08 [0.71, 0.74]	0.019

BMI, body mass index; X center, postural sway center in the medial–lateral direction; Y center, postural sway center in the anterior–posterior direction; 10MWT, 10 m walking test; HMSL, height-adjusted maximum stride length.

## Data Availability

The data presented in this study are available on request from the corresponding author. The data are not publicly available due to privacy or ethical restrictions.
